# Recurrence of Drug-Induced Lupus Secondary to Vedolizumab Use in a Patient With Crohn's Disease

**DOI:** 10.14309/crj.0000000000001270

**Published:** 2024-01-25

**Authors:** Vanessa I. Rodriguez, Akshay Mathavan, Akash Mathavan, Diana N. Rodriguez, Catalina Sanchez-Alvarez, Angela Pham

**Affiliations:** 1 Department of Internal Medicine, University of South Florida, Tampa, FL; 2 Department of Internal Medicine, University of Florida, Gainesville, FL; 3 Division of Rheumatology and Immunology, University of Florida, Gainesville, FL; 4 Division of Gastroenterology, Hepatology and Nutrition, University of Florida, Gainesville, FL

**Keywords:** drug-induced lupus, IBD, Crohn's disease, vedolizumab, infliximab

## Abstract

Drug-induced lupus is an autoimmune phenomenon characterized by the development of systemic lupus erythematosus–like clinical features after drug exposure. The entity is a clinical diagnosis. Evaluation consists of recognizing systemic lupus erythematosus–like features, identifying an appropriate causative agent, observing elevations of characteristic autoantibodies, and obtaining positive response with drug discontinuation. Vedolizumab is an anti-α_4_β_7_ antibody used in the treatment of ulcerative colitis and Crohn's disease. We report a novel case of drug-induced lupus recurrence secondary to vedolizumab use in a patient with Crohn's disease, emphasizing diagnostic evaluation, and provide a brief review of the published literature.

## INTRODUCTION

Drug-induced lupus (DIL) is an autoimmune phenomenon characterized by the development of systemic lupus erythematosus (SLE)-like clinical features after drug exposure.^1,2^ Commonly identified agents include tumor necrosis factor inhibitors.^3^ The pathogenesis of DIL is incompletely defined but includes a combination of immunogenetics and epigenetic mechanisms.^4^ There are no unified diagnostic criteria for DIL. The phenomenon is a clinical concern in patients with inflammatory bowel disease because of common use of agents known to cause DIL.^5^ Vedolizumab is an anti-α_4_β_7_ antibody used in the treatment of ulcerative colitis (UC) and Crohn's disease (CD). The monoclonal antibody has a safe therapeutic profile with low rates of infusion-related reactions or infection during treatment course.^6^

## CASE REPORT

A woman in her mid-20s with a medical history of CD presented with 1 year of arthralgias. Her treatment regimen consisted of infliximab over the past 5 years with sufficient control of previously persistent enterocutaneous fistulas and clinical remission of her CD. She described her joint pain as episodic and prominent in the bilateral hands and knees. The pain worsened throughout the day, improved with activity, and was associated with morning stiffness. The joint pain was worst immediately after her infliximab infusions. She denied fevers, weight loss, vision changes, shortness of breath, skin rash, and photosensitivity. Physical examination was notable for tenderness to palpation in the metacarpophalangeal and proximal interphalangeal joints.

The leading initial consideration was enteropathic arthropathy. However, subsequent laboratory tests were notable for elevated antinuclear antibodies (positive with 1:640 homogenous titer; reference: <1:40), anti-double-stranded DNA (anti-dsDNA) antibodies (12 IU/mL; reference: 0–9), and anti-histone antibodies (2.1 units; reference: 0.0–0.09), raising concern for DIL secondary to infliximab use. Anti-histone antibodies were not available. All other hematologic and rheumatologic laboratory results were within normal limits. Therefore, rheumatoid arthritis was excluded. Infliximab therapy was discontinued, and she started azathioprine. At a follow-up visit, the patient reported significant improvement in arthralgia, and laboratory results demonstrated normalizing anti-dsDNA levels (9 IU/mL).

Two months later, the patient initiated vedolizumab therapy. She returned after 1 month of therapy with vedolizumab reporting recurrence of arthralgias. Her symptoms appeared 1 month after discontinuing azathioprine. The joint pain was characterized similarly to that of her initial presentation. Laboratory results showed uptrending anti-dsDNA levels (11 IU/mL). Recurrence of DIL secondary to vedolizumab use was suspected. She had not been exposed to other drugs on presentation. The patient opted to continue vedolizumab and reassess her condition at a follow-up visit. Six months later, the patient reported continued mild arthralgias. After over 1 year of additional evaluations, the patient has remained on vedolizumab therapy with mild symptoms and sustained endoscopic and histologic remission.

## DISCUSSION

DIL is a challenging clinical diagnosis because of the range of phenotypic presentations—much like SLE. Hematologic abnormalities, hypocomplementemia, central nervous system involvement, or other severe manifestations are much less common in DIL when compared with SLE.^7^ Although no unified criteria for the diagnosis of DIL exist, Borchers et al^8^ proposed the following guidelines: (1) sufficient exposure to a causative agent, (2) at least 1 presenting feature consistent with SLE, (3) no previous clinical evidence suggesting SLE, and (4) resolution of symptoms within weeks to months after drug discontinuation. Characteristic autoantibody profiles are often observed in DIL, although the presence or absence of any autoantibody is not prohibitive of diagnosis. Antinuclear antibody testing is almost always positive with a homogenous pattern, and anti-histone antibodies are present in more than 90% of cases.^9^ Although anti-dsDNA antibodies are generally absent, their presence is more often observed in patients taking tumor necrosis factor inhibitors or other anticytokine therapies.^10^ A study concluded a dsDNA antibody level ≥9 U/mL is associated with clinical symptoms of DIL.^11^

Autoantibodies generated during DIL can persist for years after clinical remission of the disease, making their presence of less utility. Because of the increasingly exhaustive list of drugs associated with DIL, use of such agents after a diagnosis of DIL is relatively rare. In addition, the diagnosis of SLE with a repeat flare must be considered. Published reports of DIL recurrence are scarce.^12,13^ Moreover, to the best of our knowledge, there are only 2 published reports of vedolizumab-induced lupus, both of which were the initial presentation of the syndrome. Because of the limited number of studies and cases available, the exact mechanism of action is unclear. One patient with CD presented with acute breathlessness 2 years after vedolizumab initiation, and further evaluation confirmed DIL-pneumonitis.^14^ The second case detailed a patient with UC who presented with small joint arthralgia 3 months after vedolizumab initiation, which was later diagnosed as DIL.^15^ Symptoms resolved in both cases after drug discontinuation. Of note, both patients had previous infliximab therapy, which was discontinued because of poor clinical response. Although there are several reports on vedolizumab-induced arthritis, our patient had elevated inflammatory markers associated with DIL.

Our patient achieved symptom resolution and normalization of autoimmune markers after infliximab discontinuation, with subsequent return of joint pain 1 month after initiating vedolizumab. There were no additional clinical manifestations or laboratory values (ie, cytopenia and hypocomplementemia) to suggest enteropathic arthropathy or SLE. In addition, anti-dsDNA levels began to uptrend. No current clinical data on the utility of “trending” such markers and their relevance toward recurrence of DIL exist. We diagnosed the patient with DIL secondary to vedolizumab use. However, it is important to acknowledge that the patient met 3 of 4 DIL criteria proposed by Borchers et al.^8^ The patient decided to remain on vedolizumab therapy because she had attained clinical remission and her arthralgias were mild in severity.

This presents an interesting clinical situation in which the perceived offending agent was not discontinued, and thus, a “long-term” course of DIL was observed. Comparative studies have not shown small molecules or biologics are safer than vedolizumab.^16^ Notably, her symptoms seemed to become milder over time.

This report highlights a novel case of DIL secondary to vedolizumab use and delineates an interesting clinical course after absence of drug discontinuation. Moreover, it demonstrates the need to consider DIL in patients on vedolizumab, especially those with previous autoimmune manifestations.

## DISCLOSURES

Author contributions: VI Rodriguez performed literature review and was primarily responsible for writing the manuscript and is the article guarantor. A. Mathavan, A. Mathavan, and DN Rodriguez assisted with background research and writing of the manuscript. CS Alvarez and A. Pham revised the manuscript, provided subject matter expertise, and is responsible for its final content.

Financial disclosure: None to report.

Informed consent was obtained for this case report.
